# IFR6 EFFECT ON ORAL ADHESIONS

**DOI:** 10.51894/001c.122908

**Published:** 2024-08-30

**Authors:** Oshadi Caldera

**Affiliations:** 1 College of Osteopathic Medicine Michigan State University

## 42

### INTRODUCTION/BACKGROUND

Cleft palate is a congenital abnormality that arises due to the palatine shelves failing to fuse normally and is characterized by a hole at the primary or secondary palate. While the development can be affected by environmental and genetic factors, one gene that has been shown to be involved in normal palate development is Interferon Regulatory Gene 6 (IRF6). Specifically, it plays a potential role in the formation and/or differentiation of the periderm. The periderm is the superficial layer that covers the embryo, including the palatal shelves, and is meant to prevent adhesions of neighboring structures. In the absence of IFR6, the periderm does not form correctly creating possible adhesions that lead to abnormal development of the palate.

### OBJECTIVES/HYPOTHESIS

To distinguish the role of Irf6 in the periderm, we created transgenic mice that expressed a dominant negative mutant form of Irf6 in periderm cells, which is activated by treatment with tamoxifen.

We hypothesize that tamoxifen treatment alone would not affect palate development, and the treatment of the embryos with the transgene + tamoxifen will have abnormal palate development.

### METHODS

We collected embryos on embryonic day 13.5 and performed histological analysis of coronal sections of the oral cavity. Embryos were sham-treated or XX embryos that were tam-treated to activate the mutant transgenic allele (IRF6-DBD-ERT2-mScarlet). mScarlet is a fluorescent protein used to see if the transgene was successfully expressed in the embryos.

### RESULTS

Tamoxifen treatment alone did not cause any obvious abnormalities in the embryos. Also, we did not observe any significant abnormal oral adhesions in embryos that carried the mutant transgene and were treated with Tamoxifen.

### DISCUSSION/CONCLUSION

Although Tamoxifen is a known teratogen, the dosage used in the embryos was previously shown to be effective in inducing the activation of the ERT2 receptor, but not affect development itself. Prior studies showed that expression of the Irf6-DBD mutant transgene had a dominant negative effect on development. The lack of significant effect of the mutation is most likely explained by the transgene construct failing to express the Irf6-DBD. We are currently testing this hypothesis.

